# Association between thyroid function and regorafenib efficacy in patients with relapsed wild-type IDH glioblastoma: a large multicenter study

**DOI:** 10.1007/s11060-023-04356-w

**Published:** 2023-06-01

**Authors:** Mario Caccese, Isacco Desideri, Marta Padovan, Francesco Bruno, Giulia Cerretti, Alba Fiorentino, Luca Denaro, Franco Chioffi, Alessandro Della Puppa, Marta Maccari, Francesco Cavallin, Marina Coppola, Alice Pittaro, Roberta Rudà, Lorenzo Livi, Giuseppe Lombardi

**Affiliations:** 1 Department of Oncology, Oncology 1, Veneto Institute of Oncology, IOV-IRCCS, Padua, Italy; 2 Department of Experimental and Clinical Biomedical Sciences, Radiation Oncology, Careggi Hospital, University of Florence, Florence, Italy; 3 Division of Neuro-Oncology, Department Neuroscience, University and City of Health and Science Hospital, Turin, Italy; 4 Department of Medicine, LUM Giuseppe Degennaro University, Casamassima, Bari, Italy; 5 Academic Neurosurgery, Department of Neurosciences DNS, University of Padua, Padua, Italy; 6 Department of Neurosurgery, Padua University Hospital, Padua, Italy; 7 Neurosurgical Clinical Department of Neuroscience, Psychology, Pharmacology and Child Health, Careggi Hospital, University of Florence, Florence, Italy; 8 Independent Statistician, Solagna, Italy; 9 Pharmacy, Veneto Institute of Oncology, IOV-IRCCS, Padua, Italy; 10 Radiology Unit, Department of Imaging and Medical Physics, Veneto Institute of Oncology, IOV-IRCCS, Padua, Italy

**Keywords:** Glioblastoma, Regorafenib, Thyroid, fT3/fT4

## Abstract

**Purpose:**

Regorafenib demonstrated encouraging results in recurrent glioblastoma patients. Some studies showed that changes in circulating thyroid hormones (fT3, fT4, fT3/fT4 ratio) can be considered as prognostic factors in patients with various types of tumors. We designed this study to investigate the relationship between baseline thyroid variables and outcome in IDH-wild type GBM patients who were treated with regorafenib.

**Methods:**

This multicenter retrospective study included recurrent *IDH*-wild-type glioblastoma patients treated with regorafenib. Only patients with baseline thyroid function values (TSH, fT3, fT4, fT3/fT4 ratio) available were evaluated. RANO criteria were used to analyze neuroradiological response. Survival curves were estimated using the Kaplan–Meier method. The relationships between baseline thyroid variables (TSH, fT3, fT4, fT3/fT4) and survival (PFS, OS) were investigated with Cox regression models.

**Results:**

From November 2015 to April 2022, 134 recurrent IDH-wildtype GBM patients were treated with regorafenib and 128 of these had information on baseline thyroid function value. Median follow-up was 8 months (IQR 4.7–14.0). Objective Response Rate was 9% and Disease Control Rate was 40.9%. Median PFS was 2.7 months (95%CI 2.2–3.6) and median OS was 10.0 months (95%CI 7.0–13.0). Lower baseline TSH value in the blood was correlated with a higher rate of disease progression to regorafenib (p = 0.04). Multivariable analyses suggested a non-linear relationship between PFS (p = 0.01) and OS (p = 0.03) with baseline fT3/fT4 ratio.

**Conclusion:**

In recurrent wild-type IDH glioblastoma patients, baseline fT3/fT4 ratio showed a non-linear relationship with survival, with different impacts across the spectrum of fT3/fT4 ratio. Moreover, baseline TSH may be a predictor of regorafenib activity.

**Supplementary Information:**

The online version contains supplementary material available at 10.1007/s11060-023-04356-w.

## Introduction

Glioblastoma (GBM) is the most frequent malignant brain tumor in adults [1], and is associated with limited therapeutic options, unevitable relapse and poor prognosis [2]. Regorafenib is a small multikinase inhibitor (*VEGFR1-3, TIE2, PDGFR, FGFR, KIT, RET RAF1* and *BRAF*) [3], already approved for the treatment of several advanced or metastatic cancers [4–6]. In a previous randomized phase II trial [7], regorafenib provided some benefits in terms of overall survival (OS) compared to lomustine in recurrent glioblastoma patients after treatment according to the Stupp protocol, despite about half of the patients did not respond to regorafenib. Treatment with regorafenib resulted in grade 3–4 adverse events in 56% of treated patients; the most frequent were hand-foot skin reaction, increased lipase, and increased blood-bilirubin. Grade 1–2 hypothyroidism was experienced in 19% of patients treated in the regorafenib arm. Many studies in literature have shown that levels in circulating thyroid hormones (fT3, fT4) can be considered as prognostic factors in various clinical conditions [8–11]. Furthermore, fT3/fT4 ratio (evaluated as a surrogate for the impairment of peripheral conversion of thyroid hormones) was an independent marker of survival and frailty in elderly patients hospitalized for acute disease, even in those with normal fT3 level [8]. Few data are available on the possible prognostic effect of thyroid hormone level in patients with advanced cancer; in particular, low baseline fT3 levels appear to correlate with worse prognosis in several cancer types [12–14]. Recent studies suggested that low baseline fT3/fT4 ratio may be related to a worse overall survival (OS) and progression-free survival (PFS) in patients with advanced colorectal cancer treated with regorafenib and in patients with metastatic renal cell cancer treated with anti-VEGF drugs, regardless of the other parameters currently used in clinical practice [15–17]. Since there is no similar data regarding recurrent glioblastoma patients treated with regorafenib, we designed this multicenter, retrospective observational study to investigate the relationship between baseline thyroid variables (TSH, fT3, fT4, fT3/fT4 ratio) and survival in IDH-wild type GBM patients who were treated with regorafenib.

## Methods

We retrospectively evaluated all consecutive IDH-wildtype GBM patients who were treated with regorafenib from November 2015 to April 2022 at four high-volume Neuro-Oncological centers in Italy. Patients aged 18 years or older with histologically confirmed diagnosis of IDH wild-type glioblastoma who started treatment with regorafenib for disease recurrence/progression after chemoradiotherapy according to the Stupp protocol were included in this study. Regorafenib was administered at the standard dose of 160 mg/day for 21 days on, followed by a 7-day off, until progression or unacceptable toxicity. Dose reduction to 120 mg/day or 80 mg/day was allowed, according to the drug data sheet. Only patients for whom baseline thyroid function values were available before starting regorafenib (TSH evaluated in mIU/L, and fT3, fT4 evaluated in pmol/L) were included in the study. Patient data were collected in an anonymized database, extracting the necessary information from medical records locally. Demographic, histological, molecular, radiological characteristics of enrolled patients along with baseline thyroid function values, prior to regorafenib initiation, were recorded for all patients. According to local protocol, gadolinium brain MRI was performed at baseline and subsequently, each 2–3 months until progressive disease or when clinically indicated. Thyroid function values were evaluated locally in the participating centers at baseline and each 2–3 months during regorafenib therapy. The fT3/fT4 ratio was calculated for each individual patient. This multicenter study was coordinated by the Veneto Institute of Oncology IOV-IRCCS, was approved by the local ethics committee (EC number: 2020/154) and was conducted according to the Declaration of Helsinki.

### Statistical analysis

Continuous data were summarized as median and interquartile range (IQR), while categorical data as number and percentage. Progression-free survival (PFS) was calculated from the date of start of Regorafenib treatment to the date of disease progression or last follow-up visit. Overall survival (OS) was calculated from the date of start of Regorafenib treatment to the date of death or last follow-up visit. Survival curves were estimated using the Kaplan–Meier method. The relationships between baseline thyroid variables (TSH, fT3, fT4, fT3/fT4) and survival (PFS, OS) were investigated with Cox regression models, where thyroid variables were modeled with restricted cubic splines (with 4 knots). Multivariable analyses were conducted using Cox regression models including one baseline thyroid variable (TSH, fT3, fT4, fT3/fT4) and age, ECOG PS, tumor location, extent of surgical resection, MGMT and second surgery (only for OS) as major clinical confounding factors. Analysis of OS was restricted to patients with at least 9 months of follow-up. Effect sizes were reported as hazard ratio (HR) with 95% confidence interval (CI). All tests were 2-sided and a p-value of less than 0.05 was considered statistically significant. Statistical analysis was performed using R 4.2 (R Foundation for Statistical Computing, Vienna, Austria) [18].

## Results

### Patients

Among 134 IDH-wildtype GBM patients who were treated with regorafenib during the study period, 6 had missing information on baseline thyroid function value and were excluded from the analysis. The remaining 128 patients (92 males and 36 females; median age 60 years) were included in the analysis (Table 1). All patients underwent surgery (radical surgery in 33 patients and partial surgery in 95 patients) and received post-surgical treatment with concomitant chemotherapy and subsequent temozolomide according to Stupp protocol. Unfortunately, data on response to primary treatment were not available as the study included patients treated with regorafenib in several centers and information associated with previous treatment were not collected.Thirty-eight patients (29.7%) underwent second surgery upon relapse but data on occurrence of secondary genetic mutation after relapse were not collected.

**Table 1:** Characteristics of IDH-wildtype glioblastoma patients who were treated with Regorafenib from November 2015 to April 2022

Variable	Summary
N patients	128
Age at start of the treatment, years^a^	60 (50–65)
Time elapsed from diagnosis, years^a^	1 (1–2)
Females	36 (28.1)
Males	92 (71.9)
Tumor location	
Frontal	44 (34.4)
Parietal	26 (45.3)
Occipital/temporal	58 (20.3)
Side	
Right	54 (42.2)
Left	74 (57.8)
Type of surgery	
Radical	33 (25.8)
Non-radical	95 (74.2)
Second surgery	38 (29.7)
ECOG PS^b^	
0	15 (11.8)
1	102 (80.3)
≥ 2	10 (7.9)
MGMT methylated^b^	64 (50.4)
Baseline Corticosteroids	68 (53.1%)
TSH, mIU/L^a^	1.77 (1.09–2.65)
fT3, pmol/L^a^	4.20 (3.28–4.81)
fT4, pmol/L^a^	14.22 (12.67–16.00)
fT3/fT4, pmol/L^a^	0.29 (0.25–0.35)

Data summarized as n (%) or “a” median (IQR). “b” Data not available in one patient

### Regorafenib treatment

Patients received a median number of 2 cycles of Regorafenib (IQR 2–4). Due to toxicity, regorafenib was administered at reduced dosage (80–120 mg/die) in 50/126 patients (39.7%) during the therapy (median of previous cycles: 2.4 [IQR 1–3]). Regorafenib was administered for only 1 cycle in one patient for toxicity (skin rash CTCAE 4, fever CTCAE 2, thrombocytopenia CTCAE 2) and one patient for a rapid progression of the disease. Any grade of toxicity was reported in 119 patients (92.7%) (details in Supplementary Table 1). There were no cases of regorafenib-induced intratumoral hemorrhage. Hyperbilirubinemia CTCAE grade > 2 occurred in three patients: two died for disease progression at 5 and 21 months after Regorafenib treatment, and one was alive with no disease progression at 10 months after Regorafenib treatment. Hypertransaminasemia CTCAE grade > 2 occurred in six patients: four died for disease progression at 4–17 months after Regorafenib treatment, and two were alive with disease progression at 11–18 months after Regorafenib treatment. Unfortunately, the small number of cases prevented comparisons or further analyzes regarding the possible influence of liver function on therapeutic outcomes.

### Response to regorafenib

Among 128 patients analyzed, 122 had treatment response assessment available; in particular, 11 patients obtained a Partial Response (PR) as best response, 41 patients a Stable Disease (SD) and 70 a Progression of Disease (PD). Objective Response Rate (ORR) was 9% and Disease Control Rate (DCR) was 40.9%. Patients with disease progression had lower TSH at baseline (p = 0.04, Table 2), while not statistically significance differences were found in terms of the other thyroid variables (Table 2).

**Table 2:** Best response and baseline thyroid variables (TSH, fT3, fT4, fT3/fT4)

Variable	Patients with partial response or stable disease (n = 52)	Patients with disease progression (n = 70)	p-value
TSH, mIU/L^a^	2.26 (1.35–3.14)	1.61 (1.01–2.36)	**0.04**
fT3, pmol/L^a^	4.09 (2.99–4.81)	4.35 (3.42–4.83)	0.26
fT4, pmol/L^a^	14.01 (11.56–15.69)	14.27 (12.98–16.40)	0.51
fT3/fT4, pmol/L^a^	0.29 (0.23–0.34)	0.30 (0.25–0.35)	0.32

Data summarized as n (%) or “a” median (IQR)

### Progression-free survival and overall survival

**Fig. 1:**
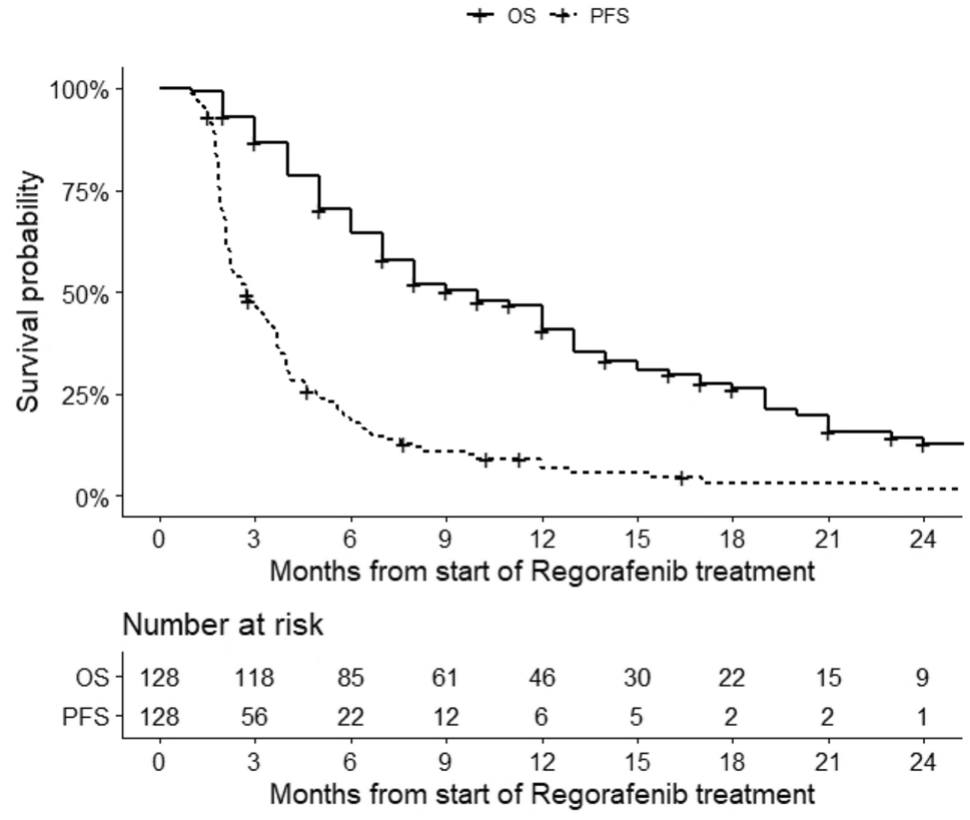
Progression-free survival (PFS) and overall survival (OS) of glioblastoma patients who were treated with Regorafenib

**Fig. 2:**
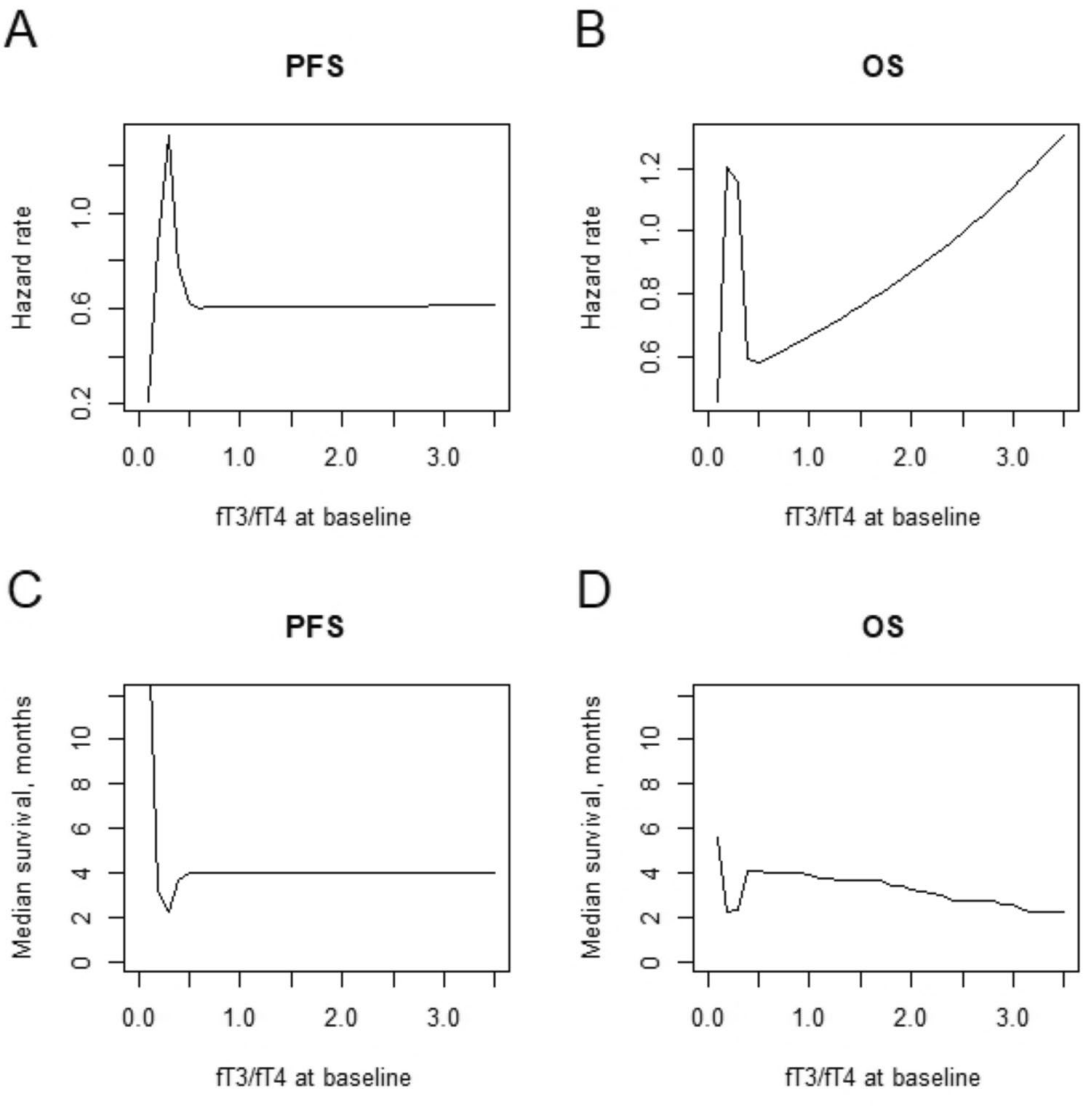
Hazard rate for PFS (**A**) and OS (**B**), and median survival for PFS (**C**) and OS (**D**), according to baseline fT3/fT4 levels modeled with restricted cubic splines in IDH-wildtype glioblastoma patients who were treated with Regorafenib

Median follow-up was 8.0 months (IQR 4.7–14.0). At the time of analysis, 118 disease progressions (92.2%) and 95 deaths (74.2%) were recorded. Median PFS was 2.7 months (95% CI 2.2–3.6), and PFS was 46.3–18.8–6.9% at 3–6–12 months respectively (Fig. 1). Median OS was 10.0 months (95% CI 7.0 to 13.0), and OS was 93.0–64.5–40.6% at 3–6–12 months respectively (Fig. 1). In all patients, univariate analysis suggested a non-linear relationship between PFS and baseline fT3/fT4 (non-linear term p = 0.008, Supplementary Table 2). When adjusting for major clinical confounding factors (age, ECOG PS, tumor location, extent of surgical resection, MGMT promoter methylation status), multivariable analysis confirmed the non-linear relationship between PFS and baseline fT3/fT4 (p = 0.01, Supplementary Table 2). The estimated hazard rate for PFS increased until fT3/fT4 of 0.3, then decreased until fT3/fT4 of 0.5, and leveled for fT3/fT4 over 0.5 (Fig. 2A). According to the points describing the shape of the curve (i.e. the points where the curve changed the slope, Fig. 2C), the estimated median PFS was lowest (2.2 months) around fT3/fT4 of 0.3, then increased to 4.0 months for fT3/fT4 of 0.5, and leveled around 4.0 months for fT3/fT4 over 0.5. In 117 patients with at least 9 months of follow-up, univariate analysis also suggested a non-linear relationship between OS and baseline fT3/fT4 (non-linear term p = 0.007, Supplementary Table 2). When adjusting for major clinical confounding factors (age, ECOG PS, tumor location, extent of surgical resection, second surgery, MGMT promoter methylation status), multivariable analysis confirmed the non-linear relationship between OS and baseline fT3/fT4 (p = 0.03, Supplementary Table 2). The estimated hazard rate for OS increased until fT3/fT4 of 0.3, then decreased until fT3/fT4 of 0.5, and finally increased for fT3/fT4 over 0.5(Fig. 2B). According to the points describing the shape of the curve (i.e., the points where the curve changed the slope, Fig. 2D), the estimated median OS was lowest (2.3 months) around fT3/fT4 of 0.3, then increased to 4.0 months for fT3/fT4 of 0.5 and decreased for fT3/fT4 over 0.5. We did not find any statistically significant associations between PFS / OS and the other thyroid variables (TSH, fT3, fT4) (Supplementary Table 2).

## Discussion

In this study, we showed a non-linear relationship between fT3/fT4 ratio at baseline and survival in recurrent glioblastoma patients treated with regorafenib. Our findings also suggested that baseline TSH value could be significantly associated with treatment response such patients. Our group collected a large and homogeneous dataset of patients with histologically confirmed diagnosis of wild-type *IDH* glioblastoma, treated with regorafenib after progression/recurrence following Stupp protocol treatment, in accordance with clinical practice. These data could open potential new scenarios in the selection of recurrent GBM patients responding to regorafenib. The rationale of our study derives from previous evidence suggesting a prognostic role of thyroid function values (and specifically fT3/fT4 ratio) both in oncological [15–17] and non-oncological settings [8]. In particular, the study by Schirripa et al. [15], demonstrated how the efficacy of regorafenib could be related to the baseline value of thyroid hormones in patients with colorectal cancer; patients were grouped according to baseline fT3/fT4 ratio tertiles of values in low, intermediate and high. Both in the exploratory cohort and in the validation cohort (expected by the study), it was demonstrated that a high value of the baseline fT3/fT4 ratio compared to intermediate and low values is significantly correlated (p = 0.003) to a better outcome in patients with metastatic colorectal cancer treated with regorafenib. To our knowledge, the association between baseline TSH value and response to regorafenib treatment in glioblastoma patients has never been reported before, and this could open new perspectives for a broad and prospective evaluation of this parameter, to improve patient selection who can benefit or not from this therapy. The alterations of the thyroid function, not caused by specific pathologies of the thyroid gland, are defined as non-thyroidal illness syndrome (NTIS), which is a very common condition in patients with different types of pathologies, often resulting in a worse prognosis and a poor response to specific treatments [8, 10, 11]. The underline mechanism is not perfectly known but may depend on various factors, including a dysregulation in the expression of receptors for thyroid hormones and thyroid hormone-binding proteins, an alteration of the metabolism of thyroid hormones themselves and/or abnormal activity of the hypothalamic-pituitary-thyroid axis [19]. Several studies have demonstrated how previous encephalic irradiation using external beam radiotherapy can cause alterations in the functionality of the hypothalamic-pituitary-thyroid axis in a pediatric population with brain cancer [20–22]. Although little information is available for adult population; A systematic review and meta-analysis evaluated that previous cerebral irradiation could be the cause of pituitary dysfunction with a prevalence of TSH alterations of 0.25 (95% CI 0.16–0.37) [23]. This impact would seem to increase over time (late effect) and, for this reason, we can hypothesize that our patients, who received a previous post-surgical radiotherapy treatment in association with temozolomide according to Stupp protocol, could have developed alterations of hypothalamic-pituitary-thyroid axis, even if not manifest from a clinical or laboratory point of view, capable of altering the fT3/fT4 ratio. It would be interesting to evaluate the dosage of hypothalamic stimulating factors, as well as the TSH and the fT3/fT4 ratio before and after radiotherapy treatment, to explore any variations and confirm our results. The circulating active amount of thyroid hormones depends on the action of some enzymes, called iodothyronine deiodinases, which are able to transform the T4 precursor into the active T3 form. There are 3 types of deiodinases (D) involved in the metabolism of thyroid hormones: deiodinases 1 (D1) and 2 (D2), responsible for the conversion of the majority of T3 and produced by the liver, kidney, and skeletal muscle; and deiodinarsise 3 (D3), which exerts its activity only at the fetal and placental tissue level but is an inactive in adults. All systemic diseases, including cancer and chronic inflammatory diseases, conditions such as cachexia and sarcopenia, or alterations in liver and kidney function, can lead to decreased D1 and D2 activity. This leads to higher T4 levels and consequent impact on fT3/fT4 ratio which can be associated with worse prognosis [8, 11, 15, 16, 19]. Historically, neuro-oncological patients, in the absence of systemic disease understood as multi-organ involvement, do not have cachexia as in other types of cancer. Sarcopenia is a combination of loss of muscle mass, strength and physical performance, which is notoriously considered a negative prognostic factor in cancer patients [24] but is poorly studied in patients with brain tumors. One of the factors influencing the qualitative and quantitative alterations of skeletal muscle tissue is certainly the prolonged use of corticosteroids, which appear to be able to promote protein degradation and inhibit protein synthesis in muscle. In particular, dexamethasone seems to be able to interrupt protein metabolism promoting skeletal muscle aging through interference with the functions of hormones such as insulin or insulin-like growth factor (IGF-1) [25, 26]. In our study, most patients were already taking corticosteroids (dexamethasone) at the time of starting treatment with regorafenib. We can hypothesize that the long-term use of corticosteroid therapy in these patients may have determined an aging of the muscle tissue, reducing the active portion of the deiodinase enzyme with a consequent impact on the fT3/fT4 ratio. Interestingly, our data suggested a non-linear relationship between fT3/fT4 ratio at baseline and survival. Such relationship followed a U-shaped curve for fT3/fT4 ratio below 0.5, suggesting improved PFS and OS with fT3/fT4 ratio departing from 0.3. Of note, the curve showed a different shape for fT3/fT4 ratio over 0.5, with a plateau for PFS and a decreasing trend for OS. We acknowledge that the small number of patients with fT3/fT4 ratio over 0.5 does not allow to draw any strong conclusions for such interval. Nonetheless, we believe that the suggested non-linear relationship between fT3/fT4 ratio at baseline and survival merits further investigations.

The strengths of this study include the novelty of the data and the homogeneity of the study sample.

To our knowledge, literature offers no data regarding the prognostic role of thyroid function values in patients with recurrent glioblastoma treated with regorafenib.

This study has also some limitations. First, the retrospective design may limit the quality and completeness of the data although only few patients were excluded due to missing information on baseline thyroid status. Second, the limited sample size suggests caution in the interpretation of the findings and might have precluded the identification of some associations. Third, the generalizability of the findings should be limited to similar patients. Fourth, it would have been interesting to evaluate how the possible development of thyroid function alterations during treatment with regorafenib could be related to survival or response to treatment in the same population, since we intend to analyze it in future studies.

In recurrent wild-type IDH glioblastoma patients baseline fT3/fT4 ratio showed a non-linear relationship with survival, with different impacts across the spectrum of fT3/fT4 ratio. Moreover, baseline TSH may be a predictor of regorafenib activity. Further studies would be necessary to establish whether a therapy with thyroid hormones and a greater control of the factors which reduce deiodination could influence the prognosis of this category of patients.

## Supplementary Information

Below is the link to the electronic supplementary material.

Supplementary file1 (PDF 62 kb)

Supplementary file2 (DOCX 16 kb)

## Data Availability

The datasets generated during and/or analysed during the current study are available from the corresponding author on reasonable request.
