# Supplementary material for: Association between thyroid function and regorafenib efficacy in patients with relapsed wild-type IDH glioblastoma: a large multicenter study
Source: J Neurooncol. 2023 Jun 1;163(2):377–83. doi: 10.1007/s11060-023-04356-w (PMC10322943; doi:10.1007/s11060-023-04356-w)
Supplement: Supplementary file 1 — Supplementary file1 (PDF 62 kb) [file 11060_2023_4356_MOESM1_ESM.pdf]

Supplementary Table 1. Toxicity of IDH-wildtype glioblastoma patients who were treated with Regorafenib.

**Variable**	**Summary**
N patients	128
N patients with toxicity	119 (92.7)
HFS:	
CTCAE grade 1	30 (23.4)
CTCAE grade 2	14 (10.9)
CTCAE grade 3	10 (7.8)
Hypertension:	
CTCAE grade 1	15 (11.7)
CTCAE grade 2	20 (15.6)
CTCAE grade 3	3 (2.3)
Asthenia:	
CTCAE grade 1	35 (27.3)
CTCAE grade 2	23 (18.0)
CTCAE grade 3	6 (4.7)
Mucositis:	
CTCAE grade 1	18 (14.1)
CTCAE grade 2	5 (3.9)
CTCAE grade 3	1 (0.8)
Dysphonia:	
CTCAE grade 1	14 (10.9)
Fever:	
CTCAE grade 1	16 (12.5)
CTCAE grade 2	6 (4.7)
Diarrhea	
CTCAE grade 1	9 (7.0)
CTCAE grade 2	2 (1.6)
Hyperbilirubinemia	
CTCAE grade 1	24 (18.8)
CTCAE grade 2	11 (8.6)
CTCAE grade 3	3 (2.3)
Hypertransaminasemia:	
CTCAE grade 1	12 (9.4)
CTCAE grade 2	6 (4.7)
CTCAE grade 3	5 (3.9)
CTCAE grade 4	1 (0.8)
Increased Amilasi/Lipasi:	
CTCAE grade 1	4 (3.1)
CTCAE grade 2	2 (1.6)
CTCAE grade 3	6 (4.7)
Skin Rash:	
CTCAE grade 1	9 (7.0)
CTCAE grade 2	5 (3.9)
CTCAE grade 3	6 (4.7)
CTCAE grade 4	1 (0.8)
Thrombocytopenia:	
CTCAE grade 1	19 (14.8)
CTCAE grade 2	2 (1.6)
CTCAE grade 3	2 (1.6)
Hypothyroidism:	
CTCAE grade 1	7 (5.4)
CTCAE grade 2	7 (5.4)

Data expressed as n (%).
