# Supplementary material for: Association between thyroid function and regorafenib efficacy in patients with relapsed wild-type IDH glioblastoma: a large multicenter study
Source: J Neurooncol. 2023 Jun 1;163(2):377–83. doi: 10.1007/s11060-023-04356-w (PMC10322943; doi:10.1007/s11060-023-04356-w)
Supplement: Supplementary file 2 — Supplementary file2 (DOCX 16 kb) [file 11060_2023_4356_MOESM2_ESM.docx]

**Supplementary Table 2.** Summary of the association between baseline thyroid variables and PFS and OS in IDH-wildtype glioblastoma patients who were treated with Regorafenib

| **Survival** | **Variable** | **Term** | **Unadjusted analysis** | **Adjusted analysis (confounding factors: age, ECOG,** **tumor location, extent of surgical resection, MGMT)** |
| --- | --- | --- | --- | --- |
| PFS | TSH | Linear term  Non-linear term | p=0.10  p=0.25 | p=0.23  p=0.23 |
|  | fT3 | Linear term  Non-linear term | p=0.65  p=0.10 | p=0.16  p=0.12 |
|  | fT4 | Linear term  Non-linear term | p=0.21  p=0.93 | p=0.55  p=0.97 |
|  | fT3/fT4 | Linear term  Non-linear term | p=0.01  p=0.008 | p=0.02  p=0.01 |
| **Survival** | **Variable** | **Term** | **Unadjusted analysis** | **Adjusted analysis (confounding factors: age, ECOG, tumor location, extent of surgical resection MGMT, second surgery)** |
| OS | TSH | Linear term  Non-linear term | p=0.35  p=0.27 | p=0.45  p=0.37 |
|  | fT3 | Linear term  Non-linear term | p=0.15  p=0.32 | p=0.29  p=0.22 |
|  | fT4 | Linear term  Non-linear term | p=0.47  p=0.36 | p=0.59  p=0.42 |
|  | fT3/fT4 | Linear term  Non-linear term | p=0.02  p=0.007 | p=0.09  p=0.03 |
